# Quality Parameters of Six Cultivars of Blueberry Using Computer Vision

**DOI:** 10.1155/2013/419535

**Published:** 2013-04-09

**Authors:** Silvia Matiacevich, Daniela Celis Cofré, Patricia Silva, Javier Enrione, Fernando Osorio

**Affiliations:** ^1^Departamento de Ciencia y Tecnología de los Alimentos, Facultad Tecnológica, Universidad de Santiago de Chile, Avenida Libertador Bernardo O'Higgins No. 3363, Estación Central, 9170022 Santiago, Chile; ^2^Departamento de Nutrición y Dietética, Facultad de Medicina, Universidad de los Andes, San Carlos de Apoquindo 2200, Las Condes, 7620001 Santiago, Chile

## Abstract

*Background*. Blueberries are considered an important source of health benefits. This work studied six blueberry cultivars: “Duke,” “Brigitta”, “Elliott”, “Centurion”, “Star,” and “Jewel”, measuring quality parameters such as °Brix, pH, moisture content using standard techniques and shape, color, and fungal presence obtained by computer vision. The storage conditions were time (0–21 days), temperature (4 and 15°C), and relative humidity (75 and 90%). *Results*. Significant differences (*P* < 0.05) were detected between fresh cultivars in pH, °Brix, shape, and color. However, the main parameters which changed depending on storage conditions, increasing at higher temperature, were color (from blue to red) and fungal presence (from 0 to 15%), both detected using computer vision, which is important to determine a shelf life of 14 days for all cultivars. Similar behavior during storage was obtained for all cultivars. *Conclusion*. Computer vision proved to be a reliable and simple method to objectively determine blueberry decay during storage that can be used as an alternative approach to currently used subjective measurements.

## 1. Introduction

Blueberries have an increasing demand for popular consumption because of their nutraceutical properties [1, 2], including their high content of phenolic compounds with a wide spectrum of biochemical activities such as antioxidant, antimutagenic, cardiovascular protection, antidiabetic, vision improvement properties, and carcinogenesis inhibition [3]. 

Blueberries are little blue fruits of the genus *Vaccinium *that have short shelf life. It has been stated that under refrigeration temperatures (0°C), the shelf life of blueberries is about 14–20 days [4, 5]. The main quality indicators of the fruit are appearance (color, size, and shape), firmness or texture, flavor (soluble solids and pH), and nutritive value [6]. The color ranges from light blue to deep black blue depending on the cultivar and the presence of an epicuticular wax, which gives its attractive appearance [4]. Color changes during storage may have a profound effect on consumer acceptability [7]. 

Consumers demand high quality fruits which are dependent on harvest methods, cultivar characteristics, postharvest handling, and storage temperatures [1]. Computer vision (CV) is a nondestructive technology used for acquiring and analyzing digital images to obtain information of heterogeneous products. It has been regarded as a valuable tool which helps to improve the automatic assessment of food quality [8, 9]. CV has been recently used in the food industry for quality and color evaluation, detection of defects, grading and sorting of fruits and vegetables, among other applications [7–11].

The objectives of this work were to study important quality factors of six different blueberry cultivars harvested in Chile under different storage conditions and to compare these cultivars as part of a comprehensive study of blueberry conservation, including the innovative technology of computer vision, which was applied in this research as a preliminary study to determine blueberry decay objectively instead of measuring it subjectively as is done nowadays.

## 2. Materials and Methods

### 2.1. Plant Material

This study was conducted during the 2009-2010 harvest season. All cultivars were donated by the Chilean Association of Exporters (ASOEX-Chile). Three blueberry cultivars (“Brigitta”, “Elliott,” and “Duke”) from Southern Highbush variety (*Vaccinium darrowii*), two cultivars (“Jewel” and “Star”) from Northern Highbush variety (*Vaccinium corymbosum*), and the cultivar “Centurion” from Rabbiteye variety (*Vaccinium virgatum*) were used. All cultivars were hand-harvested at full maturity from commercial plantations located in the central valley of the Metropolitan Region in Chile (Curacaví, Hortifrut S.A) during mid-December (“Duke” and “Jewel”), January (“Brigitta”), mid-February (“Elliott” and “Centurion”), and mid-March (“Star”) and transported to the laboratory on the same day. High quality blueberries (*n* = 50) at each storage condition were obtained by random (*n* = 10 of each clamshell) from 12 clamshells of approximately 125 mg each, presorted by hand; discarding the excessively small, soft, visually damaged, nonblue fruits, and those with the presence of pedicel and floral remains as sample set for all experiments. 

### 2.2. Storage Conditions

Blueberries (*n* = 50 at each condition) were stored at 4 and 15°C and equilibrated under different relative humidities (RH) using saturated solutions of NaCl (75% RH) and KCl (90% RH) [12] during different storage times (0, 7, 14, and 21 days). 

### 2.3. Fruit Quality Indicators

#### 2.3.1. pH and Total Soluble Solids Content

Blueberry juice was preparedfrom 5 blueberries randomly selected (in triplicate) at each storage time. The pH was measured with a pH meter (Jenway, UK) using a liquid electrode (Jenway, 924-001 model 3505) calibrated according to OMA, 1975. Total soluble solids concentration was determined by placing a drop of this blueberry juice (1 mL) on a calibrated portable refractometer (0–32 °Brix, RHB-32ATC). The mean and standard deviation of three replicates were recorded and expressed as °Brix.

#### 2.3.2. Fruit Size and Form

The equatorial and polar diameter of each blueberry was measured with a digital caliper (Bull Tools, USA), and the roundness index (RI) was determined from (1). Mean and standard deviation of all blueberries (*n* = 50) measured at each storage condition were reported. These parameters were also obtained from image analysis, correlating linearly with experimental data (*R* ^2^ = 0.998).

(1) $$\begin{array}{c} \text{RI}=\frac{\text{polar}\;\text{diameter}}{\text{equatorial}\;\text{diameter}}. \end{array}$$

#### 2.3.3. Water Content and Fruit-Dehydrated Percentage

Water content was gravimetrically determined using an analytical balance (Mettler Toledo, Switzerland). Twelve blueberries (in triplicate) were dried for 24 h in an oven (Wiseven, Korea) at 105°C until constant weight. Water content was expressed as wet basis percentage (g water/100 g wet sample).

**Figure 1:**
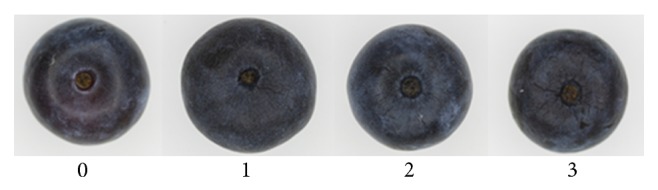
Fruit dehydration degree visually observed following the norms for quality of fresh blueberries from Chilean Blueberry Committee (CBBC, 2011).

Dehydrated fruit percentage was evaluated visually using the photographies obtained by computer vision, taking into account the different degrees of fruit dehydration (Figure 1) following the norms for quality of fresh blueberries from Chilean Blueberry Committee [13]. Fruits with dehydration degree of 2 or 3 were counted as dehydrated fruit.

### 2.4. Image Analysis

#### 2.4.1. Color

**Figure 2:**
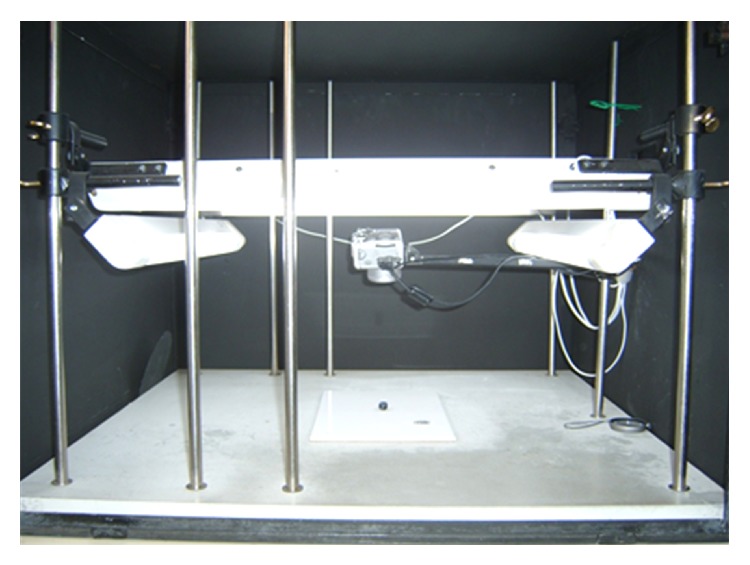
Computer vision system. Elements distribution in the digital image acquisition.

Digital images of each blueberry (of two opposite sides) were taken at each storage time in order to obtain the surface color of the fruit using a computer vision system (Figure 2), which consisted of a black box with four 18 W natural light tubes (D65, Philips) and a digital camera (Canon 10 MP, PowerShot G4) placed in a vertical position 22.5 cm from the samples (camera lens angle and lights at 45°) [9]. All images were obtained under the same conditions; the camera was remotely controlled by ZoomBrowser software (v.6.0, Canon). Surface color data were measured in the CIEL ^*^ *a* ^*^ *b* ^*^ space and image analysis was performed with the Balu Toolbox in Matlab software (v7) [8]. The camera parameters and Balu software were calibrated using 30-color charts with a Minolta colorimeter. Therefore, *L* ^*^ *a* ^*^ *b* ^*^ values obtained from image analysis were equal to the values from the colorimeter.

Color variation, CIEDE2000, or Δ*E*_00_, is regarded as the best uniform color difference model coinciding with subjective visual perception, which can reflect the color difference between two images. The color variation (Δ*E*_00_, (2)) during storage time was determined using the formulas which include the concepts of chroma (*C* ^*^, (3)) and hue (*h*′, (4)) for *L* ^*^ *a* ^*^ *b* ^*^ values [14]. The color grade differences (Δ*E*_00_) between two samples (1, 2) were determined as follows: the perception of color differences was taken as imperceptible if Δ*E*_00_ < 1.5, noticeable if Δ*E*_00_ < 3, and appreciable if Δ*E*_00_ < 6 [15].

(2) $$\begin{array}{c} {\Delta E}_{00}=\;\sqrt{{\left(\frac{{\Delta L}^{\prime }}{K_{L}S_{L}}\right)}^{2}+{\left(\frac{{\Delta C}^{\prime }}{K_{C}S_{C}}\right)}^{2}+{\left(\frac{{\Delta H}^{\prime }}{K_{H}S_{H}}\right)}^{2}+R_{T}\left(\frac{\Delta C^{\prime }}{K_{C}S_{C}}\right)\left(\frac{{\Delta H}^{\prime }}{K_{H}S_{H}}\right)}, \end{array}$$

(3) $$\begin{array}{c} C_{i,ab}^{*}=\sqrt{{\left(a_{i}^{*}\right)}^{2}+{\left(b_{i}^{*}\right)}^{2}},\;i=1,2, \end{array}$$

(4) $$\begin{array}{c} \;h_{i}^{\prime }=\tan^{-1}\left(\frac{b_{i}^{*}}{a_{i}^{\prime }}\right),\;i=1,2, \end{array}$$

where

(5) ai′=(1+G)ai∗, i=1,2,G=0.5(1−Cab∗−2Cab∗−7+257),Cab∗−=(C1,ab∗+C2,ab∗)2,Ci′=(ai∗)2+(bi∗)2, i=1,2,∆L′=L2∗−L1∗,∆C′=C2∗−C1∗,∆h′=h2′−h1′,∆H′=2C1′C2′sin(Δh′2),L′−=(L1∗+L2∗)2,C′−=(C1′+C2′)2,h′−=(h1′+h2′)2,SL=1+0.015(L′−−50)220+(L′−−50)2,SC=1+0.045C′−,    SH=1+0.015C′−T,T=1−0.17cos(h′−−30)+0.24cos(2h′−)+0.32cos(3h′−+6)−0.20cos(4h′−−63),RT=−sin(2Δθ)RC,∆θ=30exp{−[(h′−−275°)25]2},RC=2C′−7(C′−7+257),KL=Kh=KC=1

(see [16]).

#### 2.4.2. Fungal Presence

Fungal presence percentage was obtained by image analysis by computer vision, taking as positive blueberry when fungal filaments were visually observed according to (6):

(6) $$\begin{array}{c} \%\text{Fungal}\;\text{presence}=\frac{\text{No}.\;\text{of}\;\text{positive}\;\text{blueberries}}{\text{No}.\;\text{of}\;\text{total}\;\text{blueberries}}\times 100. \end{array}$$

In order to validate the fungal presence percentage visually observed by image analysis, fungal filaments from fruits were extracted by immersion of fruits in 5 mL of distilled water for 1 min with manual agitation. Turbidity of extracted aqueous samples was measured by absorbance at 720 nm. A linear correlation of fungal presence on fruits that measured both turbidity and image analysis (*R* ^2^ = 0.995) was obtained. Pearson correlation coefficient was 0.99, indicating a good positive correlation between the values reported by absorbance and image analysis using (6) [7]. 

### 2.5. Statistical Analysis

Statistical analysis was made by analysis of variance (ANOVA) and Tukey's posthoctest, considering significant differences if *P* ≤ 0.05. Pearson's correlation coefficient (*P*) was also calculated.

## 3. Results and Discussions

In first place, a characterization of quality indicators of six cultivars was determined at initial time (fresh blueberries) to obtain possible differences among cultivars. Then, a characterization of each quality indicator was made during storage time under different storage conditions in order to obtain the shelf life and the behaviour of blueberries under the studied storage conditions.

### 3.1. pH and**  **°Brix

All evaluated cultivars were from the same field conditions (Summer 2009-2010) in order to avoid the influence of growing conditions such as soil, pH, and water that could affect the pH of the fruits [17]. The pH and °Brix obtained from each fresh cultivar are shown in Table 1.

The initial pH (fresh fruits) founded in “Duke” (pH = 3.74 ± 0.11) was higher than other blueberry cultivars, while the lowest value was obtained for “Elliott” (pH = 2.9 ± 0.06). The reason for these differences would be the citric acid concentration present in each cultivar, which also depends on genetic differences [17]. The pH values obtained for all the cultivars were in the range of 2.75–3.81, in agreement with previous literature reports for other different blueberry cultivars [18]. 

The highest °Brix values were found in “Centurion” (°Brix = 13.9 ± 0.5), and “Elliott” (°Brix = 13.5 ± 0.5) and the lowest value was found for “Jewel” (°Brix = 11.9 ± 0.4). These results are in agreement with the expected range of 11.2–14.3 °Brix reported for other blueberry cultivars [18, 19]. 

**Figure 3:**
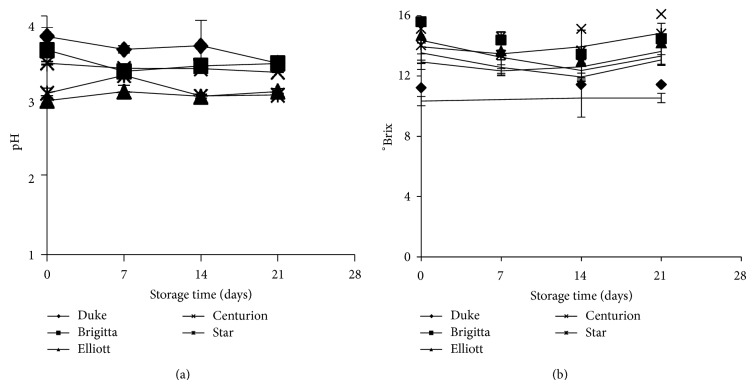
pH and °Brix variations of different cultivars for different storage times at 4°C and 90% RH.

Although pH and °Brix values were different among cultivars of fresh fruits, these values remained constant with respect to the initial values (*P* > 0.05) during the storage time, regardless of storage conditions (Figure 3). It has been reported that the increasing pH in the fruit is due to maturing time on the plant and also to dehydration during postharvest storage [1, 20]. Therefore, the constant values of pH and °Brix during postharvest storage obtained in this study could be an indicative that no significant dehydration occurred during evaluated storage conditions.

### 3.2. Fruit Size and Shape

The equatorial diameter and roundness index (RI, (1)) of each fresh cultivar are shown in Table 1. All the cultivars presented an equatorial diameter greater than 1 cm, which is required to satisfy Chilean export specifications. Therefore, these blueberries were not considered as low calibre [13]. However, the percentage variation of the equatorial diameter of all the cultivars (50–60%) was higher than 3%, which is established for export standards.

The roundness index (RI) indicated that “Centurion” had a significant RI difference (*P* < 0.05) compared to the other cultivars, presenting a more spherical shape (0.95 ± 0.03), while “Elliott” presented the smallest RI (0.72 ± 0.01).

**Figure 4:**
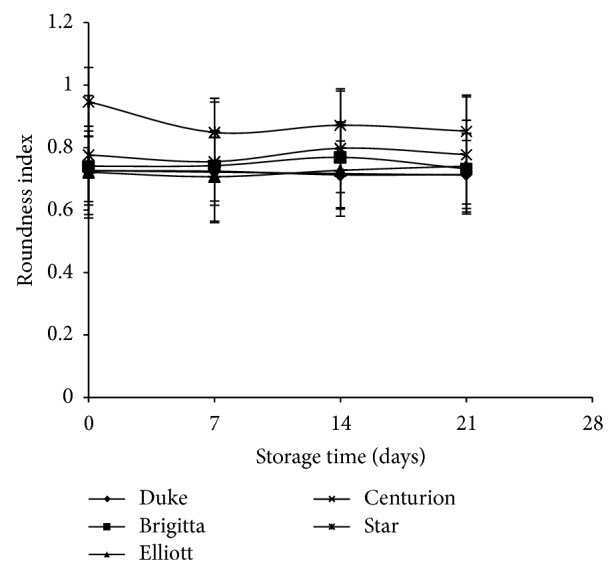
Roundness index (RI) of different cultivars over time at 4°C and 90% RH.

After harvest, fruit size could be altered by both water content, which is kept within in the cell by osmotic forces, and degradation of peptic substances, which weakens the cell walls. Consequently, the fruits cannot retain their shape and integrity [21]. However, under the two storage conditions studied (75% and 95% RH; 4 and 15°C) no significant differences (*P* > 0.05) in both round index (RI) and equatorial diameter were found among the different cultivars (Figure 4). These results indicated that neither significant dehydration nor pectin degradation occurred during storage time under both storage conditions studied.

### 3.3. Water Content and Dehydrated Fruits

The water content of each fresh cultivar is shown in Table 1. The cultivar with the highest water content value was “Brigitta” (87.5 ± 0.7% wb), and the lowest water content value was obtained for “Jewel” (79.9 ± 0.8% wb). 

At the storage conditions, no significant differences (*P* > 0.05) were found in water content in each cultivar during the whole storage period, which indicates that the blueberries did not undergo significant dehydration (*P* > 0.05) during storage under the controlled temperature and humidity.

The percentage of fruits with presence of surface dehydration at final storage time (21 days), calculated using image analysis following dehydration degree of Chilean Blueberry Committee [13], is presented in Table 2. The results showed that although water content values are constant among storage time for all cultivars, the percentage of fruits with surface dehydration degree higher than 2 on all evaluated cultivars increased with high temperature and low relative humidity, as expected.

Therefore, the results showed that the quality parameters of pH, °Brix, shape, roundness index, and water content of fresh blueberries are different among the evaluated six cultivars hand-harvested in Chile. However, the storage behaviour was similar between them independently of temperature and humidity conditions, indicating that in the selected storage conditions no significant differences (*P* > 0.05) were obtained during storage time. Regarding these important quality parameters, which remained constant during storage time, could not indicate deteriorative changes on the evaluated storage conditions. However, a surface dehydration of cuticle of fruits was detected visually using image analysis. This is an important approach to define a damage pattern with different quality degree levels, which can be designed by automatic classification algorithms to be implemented in the industry, reducing overall batch rejections for the market.

### 3.4. Color

The use of image analysis using computer vision allowed differentiating the blueberry color of different cultivars at various storage times. 

The lightness (*L* ^*^ value) of the blueberry surface showed significant differences (*P* < 0.05) in the initial color of fresh “Duke” cultivar compared to the other cultivars, among which no significant differences (*P* > 0.05) were found (Table 1). This *L* ^*^ value for “Duke” could not be associated with a lower presence of epicuticular wax on this fruit's surface because the percentage of epicuticular wax (30 ± 5%) was similar among the cultivars, as expected [7]. Similar results (28 ± 8%) were found by Matiacevich et al. [7] comparing “Duke”, “Jewel,” and “Elliott” cultivars. 

**Figure 5:**
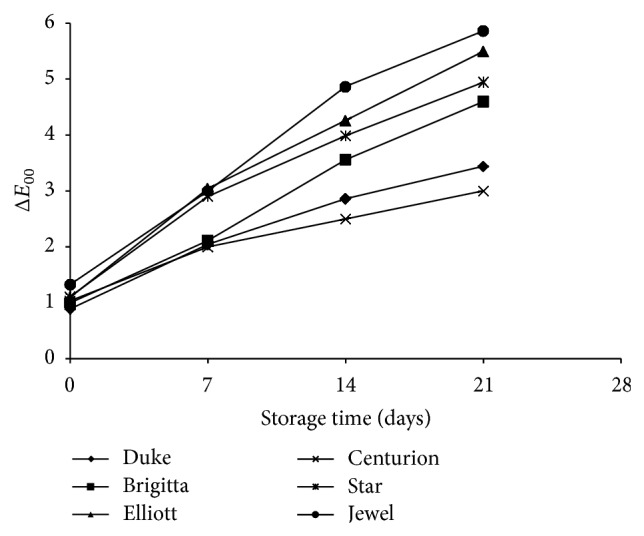
Color change (Δ*E*_00_) of different cultivars over storage time at 4°C and 90% RH.

Color change during storage, observed as Δ*E*_00_ (2), showed that the behaviour of each cultivar does not differ significantly (*P* > 0.05) as a function of storage time at 4°C and 90% RH (Figure 5). At 7 days of storage, the color changes are imperceptible for all cultivars. However, appreciable color differences (Δ*E*_00_ > 3) were obtained for “Jewel”, “Elliott”, “Star,” and “Brigitta” at final storage time of 21 days. Only “Duke” retained a noticeable range of color change value at the end of the storage period.

The observed behaviour in Figure 5 was similar to those fruits under the other storage conditions, indicating that the color variation was mainly due to differences among cultivars and not to storage temperature and relative humidity conditions. The color change was appreciable from the initial blue to a red color, which was observed by changes in both *a* ^*^ and *b* ^*^ values, indicating senescence of the fruit. The *a* ^*^ values for all fresh blueberries analysed in this study were obtained in the range from −5 to 5 and *b* ^*^ values from −10 to 5, as in agreement with those found for the same cultivars [7]. However, these values increased during storage time showing a range for *a* ^*^ of 0–12 and for *b* ^*^ from −2 to 6. As a function of storage time, the color change occurred in more than 90% of the blueberries for all cultivars, except for “Centurion”, where the color change occurred in around of 75% of the fruits. 

These results showed the importance of color fruit as an indicative of deterioration of blueberry quality, which was determined using image analysis.

### 3.5. Fungal Presence

As expected, fungal presence obtained through image analysis by computer vision using (6) was affected significantly (*P* < 0.05) by storage conditions: temperature and time. Figure 5 shows that fruits with fungal presence increased with increasing storage temperature and time for all cultivars analyzed, showing a lower growth kinetic at 4°C (2%) than at 15°C (up to 14%) at both RHs after 21 days.

It is important to note that the behavior under each storage condition was different for all cultivars, where “Jewel” was more susceptible to fungal growth, emphasizing that the development of *Botrytis* in blueberries could be due to the presence of fungi in the fruit in the initial phase of the study as natural inoculums, and it was not exposed intentionally or inoculated during its storage. Therefore, the differences between cultivars may mainly be due to differences of initial inoculums obtained in the field and not to different genomic susceptibility between cultivars.

The color parameter, lightness (*L* ^*^) increased during storage time (Figure 7). This increase was related to fungal presence in all cultivars, which was attributed to *Botrytis cinerea* identified taxonomically [22]. Pearson correlation coefficient between lightness and fungal presence was higher than 0.9 for all cultivars, indicating that total lightness increased principally due, the characteristic white-gray color of *Botrytis *filaments [22]. 

Therefore, the results showed that color changes outside the initial color range of each cultivar are an important quality factor to define another damage pattern, which could be measured by image analysis and, therefore, is possible for designed automatic algorithms using computer vision.

### 3.6. Shelf Life

The shelf life of blueberries is based mainly on visual choice by consumers during its consumption, where the conditions that may change during storage time are the most important ones to be taken into account. Therefore, the factors considered on determination of shelf-life in this study were fungal presence and color change.

Since the determination of shelf-life is a subjective parameter, its determination was based on the occurrence of some of the following conditions: (i) color change from blue to red in more than 45% of the samples and/or (ii) fungal presence higher than 2% due to the only presence of fungal filaments is unacceptable by the consumers [7].

**Figure 6:**
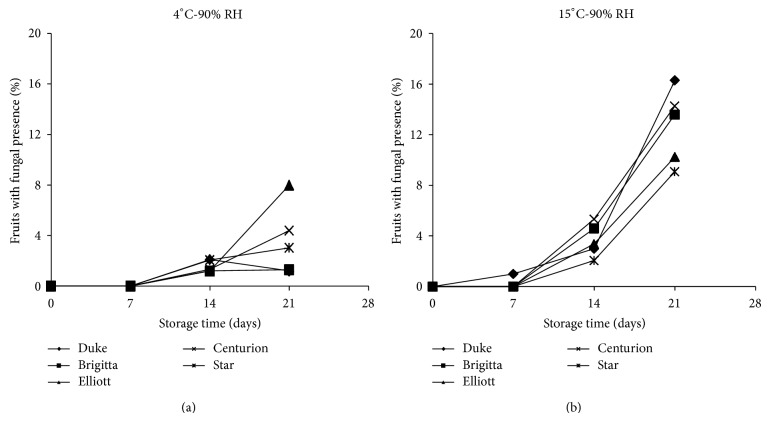
Percentage of fruits with fungal presence (6) in six blueberry cultivars stored under different storage conditions (4 and 15°C and 90% RH).

**Figure 7:**
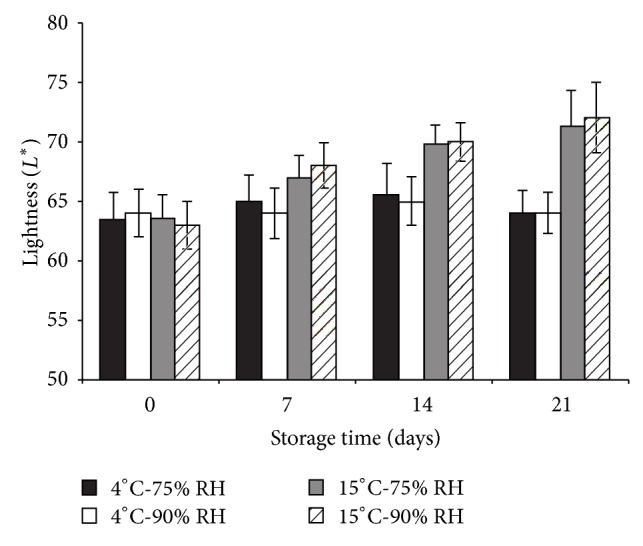
Total lightness (*L* ^*^) of “Brigitta” at different temperatures (°C) and relative humidities (RH) of storage conditions. Similar behavior was observed for other cultivars.

According to information delivered by blueberry producers and used in this research, the shelf-life of different cultivars at 90% RH and 0–4°C is 20 days. However, experimental data indicated that shelf life was 14 days for all cultivars, principally due to fungal presence higher than 2%, as shown in Figure 6.

## 4. Conclusions

Quality parameters (pH, °Brix, shape, water content, and color) were different among fresh cultivars, as expected, due principally to genetic differences between them and these values did not changed during the evaluated storage conditions. Other parameters depended on storage conditions, as expected such as color changes from blue to red by time in all conditions, surface dehydration, and fungal presence, which both increased principally with temperature and time. However, fungal presence was considered the most important quality parameter to determine a shelf-life due to its unacceptability by consumers. 

Despite the differences on temperature and humidity of the storage conditions, the shelf life (taking into account more than 2% of fruits with presence of fungal filaments) was calculated as 14 days for all cultivars independently of the storage conditions. 

Moreover, the innovative technology of computer vision applied in this research was a useful tool to determine blueberry decay such as color, surface dehydration, and fungal presence, in an objective manner instead of measuring it subjectively as is done nowadays.

Practical applications of the results obtained in this study are related to the knowledge of the important quality factors from six different blueberry cultivars harvested in Chile under different storage conditions, as part of a comprehensive study of blueberry conservation. Computer vision could be used as an approach to obtain damage patterns that define different quality levels of blueberries. This technology allows designing automatic classification algorithms to be implemented in the industry based on its simplicity, allowing also the analysis of heterogeneous materials such as fresh fruits. 

## Figures and Tables

**Table 1:** Quality parameter differences between fresh cultivars.

Cultivar	pH	°Brix	RI^1^	Water content (% wb)^2^	*L* ^*^^3^
“Duke”	3.74 ± 0.11^(a)^	10.33 ± 0.31^(a)^	0.73 ± 0.01^(a)^	86.50 ± 0.52^(a)^	64.96 ± 2.82^(a)^
“Brigitta”	3.57 ± 0.17^(a,c)^	12.70 ± 0.53^(a,b)^	0.74 ± 0.01^(a)^	87.59 ± 0.72^(a)^	71.49 ± 1.97^(a)^
“Elliott”	2.94 ± 0.06^(b)^	13.20 ± 0.10^(b)^	0.72 ± 0.01^(a)^	83.46 ± 0.79^(b)^	72.85 ± 5.20^(a)^
“Centurion”	3.41 ± 0.02^(c)^	13.93 ± 1.50^(b)^	0.95 ± 0.03^(b)^	82.36 ± 0.83^(b)^	71.85 ± 1.94^(a)^
“Star”	3.03 ± 0.06^(b)^	12.93 ± 0.11^(b)^	0.78 ± 0.01^(c)^	83.97 ± 0.51^(b)^	71.78 ± 3.00^(a)^
“Jewel”	3.50 ± 0.10^(c)^	11.90 ± 0.38^(a)^	0.82 ± 0.02^(d)^	79.86 ± 0.85^(c)^	72.20 ± 2.50^(a)^

Different superscript letters for the same column indicate values to be significantly different (*P* < 0.05).

^1^RI: roundness index (1).

^2^% wb means wet basis percentage.

^3^ *L* ^*^: color parameter of lightness.

**Table 2:** Percentages (%) of dehydrated fruits take into account visually surface dehydration at different storage conditions of temperature (°C) and relative humidity (RH) after 21 days of storage.

Cultivar	4°C 75% RH	4°C 90% RH	15°C 75% RH	15°C 90% RH
“Duke”	12	10.7	21	19
“Brigitta”	5	2.7	35.1	33.3
“Elliott”	20.1	15.5	35.7	35.4
“Centurion”	20	18.7	40.2	39.6
“Star”	37	36	38.1	38
“Jewel”	15.5	12.5	38.1	37.5
